# Challenges in Managing Recurrent Small Bowel Obstruction in a Patient With Concurrent Hepatic Dysfunction and Alcohol Use Disorder: A Multidisciplinary Approach

**DOI:** 10.7759/cureus.85139

**Published:** 2025-05-31

**Authors:** Kaan Sevgi, Logan Enman, Francisco Lucero, Sinan Nazif Aran

**Affiliations:** 1 School of Medicine, Kansas City University of Medicine and Biosciences, Kansas City, USA; 2 Department of Internal Medicine, University of Miami/HCA Florida, Coral Gables, USA; 3 Department of Family Medicine, Demiroglu Science University, Istanbul, TUR

**Keywords:** alcohol use disorder, gastrointestinal motility, hepatic dysfunction, multidisciplinary care, small bowel obstruction

## Abstract

This case report describes a 63-year-old Black man with recurrent small bowel obstruction (SBO), persistent abdominal pain, and hepatic dysfunction, set against a complex background of idiopathic peripheral neuropathy, chronic hepatomegaly, an indeterminate adrenal mass, and long-term alcohol use. Imaging revealed dilated bowel loops with partial obstruction, while laboratory findings showed elevated liver enzymes indicative of hepatic impairment. His clinical course highlighted the multifactorial nature of SBO, likely influenced by anatomical disruptions, hepatic pathology, and systemic metabolic factors, including potential adrenal involvement. The case emphasizes the importance of a multidisciplinary approach integrating gastroenterology, hepatology, and endocrinology to manage such intricate presentations. It also underscores the role of chronic alcohol use in exacerbating gastrointestinal and hepatic conditions, advocating for a holistic treatment strategy to optimize outcomes.

## Introduction

Small bowel obstruction (SBO) is a prevalent surgical emergency, accounting for approximately 15% of acute abdomen cases in adults. Its management becomes increasingly complex when accompanied by comorbidities such as liver disease and chronic alcohol use. Alcohol consumption is a well-established risk factor for liver disease, including conditions like alcoholic hepatitis and cirrhosis, which can significantly complicate the clinical course of SBO [1]. Chronic alcohol use also leads to gastrointestinal motility disorders, further complicating SBO management [2].

The interplay between alcohol-induced liver disease and gastrointestinal motility disorders is multifaceted. Alcohol disrupts gut microbiota, increasing intestinal permeability and systemic inflammation, which exacerbates liver injury and impairs gastrointestinal function [3]. Additionally, alcohol-induced neuropathy can affect the enteric nervous system, leading to dysmotility and increasing the risk of bowel obstruction [4].

In patients with liver cirrhosis, the risk of developing SBO is heightened due to factors like portal hypertension and ascites, which can result in bowel wall edema and impaired motility [5]. Cirrhotic patients often have altered immune responses, making them more susceptible to infections and complications during SBO management [6].

The management of SBO in patients with liver disease and alcohol use disorder requires a comprehensive approach. Supportive care, including fluid resuscitation and electrolyte correction, is essential. Pharmacologic interventions may involve prokinetic agents to enhance gastrointestinal motility, though caution is necessary due to potential hepatotoxicity [7]. Monitoring of liver function tests (LFTs) and nutritional status is crucial, as malnutrition is common in this patient population and can negatively impact outcomes [8].

A multidisciplinary approach involving gastroenterologists, hepatologists, surgeons, and nutritionists is often necessary to address these patients' complex needs. Early recognition and management of alcohol withdrawal syndrome are essential, as withdrawal can precipitate complications and hinder recovery [9]. Addressing the underlying alcohol use disorder through counseling and rehabilitation is vital to prevent recurrence and improve long-term outcomes [10].

Chronic alcohol use impacts multiple metabolic pathways, impairing protein synthesis, mTOR signaling, and hepatic function, contributing to a more severe clinical course in patients with SBO and liver disease [11,12]. Alcohol use is also associated with alterations in gut-liver signaling, influencing liver pathology through inflammatory mechanisms [13-15].

Chronic alcohol intake disrupts the gut-liver axis, increasing intestinal permeability and inflammatory responses that contribute to liver injury [16-18]. Alcohol-related liver disease (ALD) progresses through stages, from fatty liver to hepatitis, fibrosis, and cirrhosis, with cirrhosis predisposing patients to complications like ascites and impaired bowel motility, which increase SBO risk [19,20].

## Case presentation

A 63-year-old Black man with a history of chronic alcohol use disorder presented to the emergency department (ED) with worsening abdominal discomfort, nausea, early satiety, constipation for one week, and escalating abdominal pain. His past medical history included idiopathic peripheral neuropathy, chronic hepatomegaly, an adrenal gland mass of indeterminate origin, colonic polyps, and hypertension. The patient reported a 20-pack-year smoking history and daily consumption of 3-5 vodka shots. Current medications include atenolol, gabapentin, lactulose, pantoprazole, tamsulosin, thiamine, and varenicline.

On initial evaluation, he was afebrile with a blood pressure of 113/69 mmHg, heart rate of 98 beats per minute (bpm), and respiratory rate of 18 bpm. He appeared in no acute distress but exhibited a distended abdomen with upper quadrant tenderness and voluntary guarding. Laboratory results were notable for leukocytosis, mild anemia, and elevated liver enzymes consistent with underlying hepatic dysfunction, likely secondary to chronic alcohol use. A summary of his laboratory findings is shown in Table 1.

**Table 1:** Initial laboratory findings on admission

Test	Result	Reference range	Unit
White blood cell (WBC) count	15.7	4.0-11.0	×10⁹/L
Hemoglobin (HGB)	12	13.5-17.5	g/dL
Aspartate aminotransferase (AST)	50	10-40	U/L
Alkaline phosphatase (ALP)	185	40-130	U/L
Total bilirubin	1.6	0.1-1.2	mg/dL

**Figure 1:**
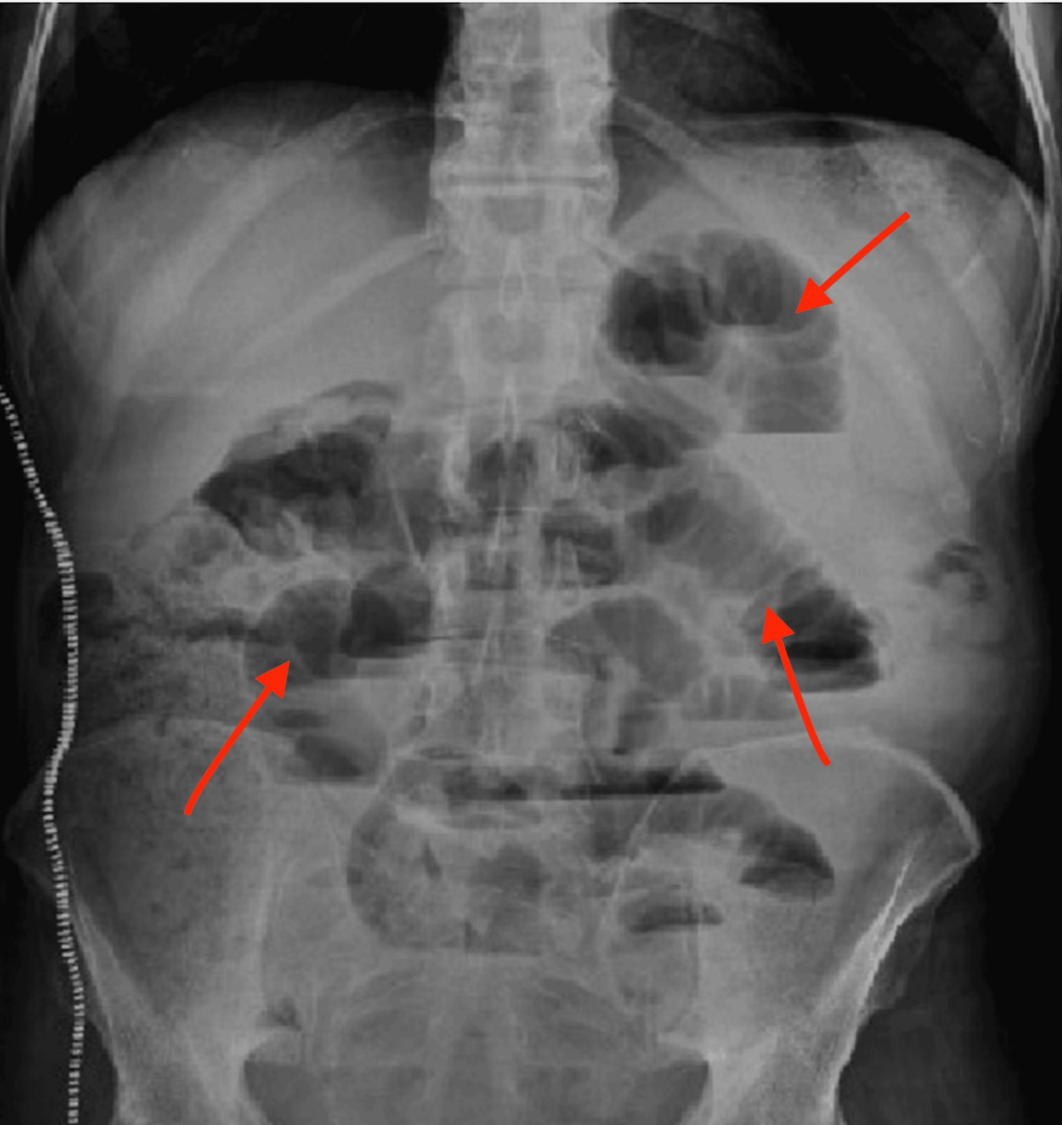
Upright abdominal X-ray showing multiple distended loops of small bowel with air-fluid levels, consistent with partial small bowel obstruction The arrows indicate the areas of bowel dilation.

**Figure 2:**
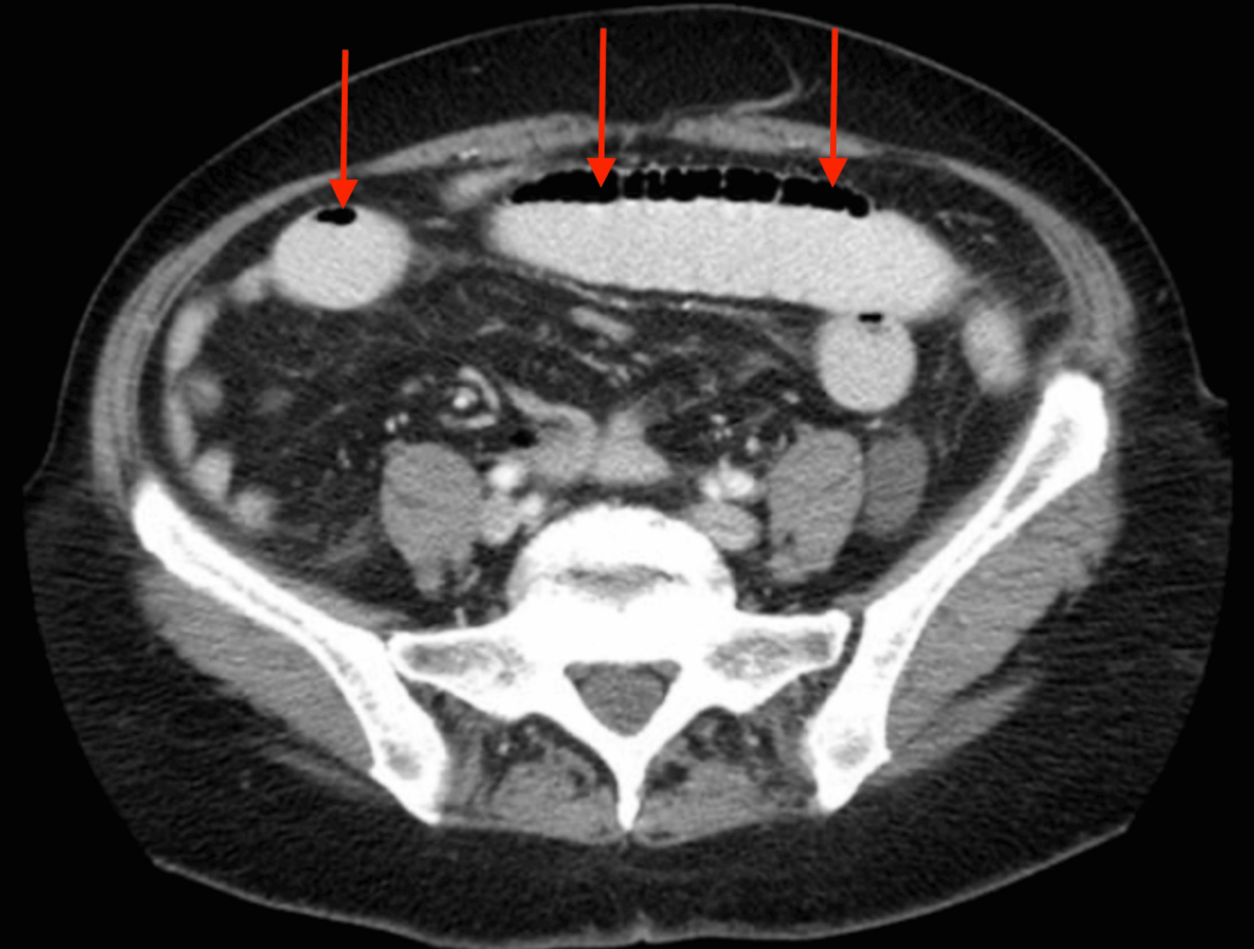
Axial CT scan of the abdomen demonstrating dilated small bowel loops with fluid-filled segments and a paucity of colonic gas, indicative of partial obstruction. Hepatic steatosis and trace ascites are also visible The arrows highlight the dilated bowel segments.

Abdominal radiography revealed dilated small bowel loops and multiple air-fluid levels consistent with a partial SBO (Figure 1). Follow-up computed tomography (CT) imaging confirmed the diagnosis, showing marked small bowel dilation without evidence of a discrete transition point, consistent with partial SBO, and additional findings of hepatic steatosis and trace ascites (Figure 2).

Initial management consisted of bowel rest, nasogastric tube decompression, intravenous fluid resuscitation, and non-narcotic analgesia. A bowel regimen of lactulose, senna, and docusate was continued, along with thiamine and folate supplementation to address suspected alcohol-related nutritional deficiencies. Given the concern for bacterial translocation due to compromised gut integrity in the context of liver dysfunction, empirical intravenous antibiotics (ceftriaxone and ampicillin-sulbactam) were administered.

Throughout hospitalization, the patient experienced intermittent abdominal pain and nausea, though without signs of peritonitis or strangulation. Repeat imaging showed persistent but non-progressive findings of SBO. Gastroenterology and general surgery consultations recommended ongoing conservative management. By hospital day 6, the patient demonstrated clinical improvement, including the return of bowel function, tolerance of a clear liquid diet, and resolution of abdominal symptoms. He was discharged in stable condition with recommendations for outpatient follow-up, alcohol cessation counseling, and coordinated multidisciplinary care to address the interplay of hepatic dysfunction, gastrointestinal motility, and nutritional status.

This case illustrates the complex interplay between chronic liver disease and recurrent SBO, emphasizing the importance of early recognition, conservative management, and interdisciplinary collaboration to optimize outcomes in patients with multifactorial gastrointestinal pathology.

## Discussion

This case exemplifies the intricate challenges encountered when managing recurrent SBO in patients with coexisting hepatic dysfunction and alcohol use disorder. The patient’s presentation, marked by hepatomegaly, constipation, and abdominal pain, required a tailored approach that took into account the numerous physiological disruptions caused by chronic alcohol consumption and liver disease. Alcohol is a potent hepatotoxin that leads to a range of liver-related complications, including hepatomegaly and hepatic steatosis, and ultimately progresses to cirrhosis and liver failure if left unchecked [3]. These hepatic changes have wide-reaching implications for the management of SBO, as liver disease directly impacts drug metabolism, immune response, and gastrointestinal motility, which are essential factors in SBO management [4].

Impact of chronic alcohol use on liver and drug metabolism

Chronic alcohol consumption leads to significant hepatic impairment, which complicates the metabolic processing of medications. The liver’s reduced ability to metabolize drugs is especially challenging in SBO cases, where supportive treatments may include antibiotics, analgesics, and possibly prokinetics. In cirrhotic patients, hepatotoxic risks associated with these medications are magnified due to the liver’s diminished ability to filter toxins and process drugs effectively. For instance, prokinetic agents often used to manage gastrointestinal (GI) motility disorders in SBO must be administered cautiously, as they can exacerbate liver damage in patients with hepatic impairment [7]. Medications need to be selected and dosed carefully, often requiring lower-than-standard doses and close monitoring of LFTs to avoid hepatotoxicity and adverse effects [6]. This limitation complicates the management strategy for SBO in such patients and necessitates a multidisciplinary team to ensure a balanced therapeutic approach.

The gut-liver axis and immunological vulnerability

The pathogenesis of ALD underscores the role of the gut-liver axis in exacerbating hepatic dysfunction. Alcohol disrupts gut microbiota and increases intestinal permeability, a phenomenon known as “leaky gut.” This disruption allows bacterial endotoxins to enter the portal circulation, causing a cycle of inflammation and hepatic damage as these toxins reach the liver [4,13]. The systemic inflammatory response elicited by these endotoxins contributes to hepatic inflammation, accelerates fibrosis, and increases the patient’s susceptibility to complications like sepsis during SBO episodes. Patients with cirrhosis or severe liver disease are particularly vulnerable to infections such as spontaneous bacterial peritonitis, which can further exacerbate SBO symptoms and lead to severe complications [2,9].

In addition to predisposing patients to infections, this immune vulnerability impacts surgical and non-surgical treatment options for SBO. When considering surgical intervention, the altered immune response and increased risk of infection in patients with cirrhosis may result in higher postoperative morbidity and mortality rates. For this reason, a conservative, non-surgical approach to SBO, focusing on bowel rest, fluid management, and decompression, is often preferred initially [19].

Managing alcohol withdrawal in hospitalized patients

For patients with alcohol use disorder, hospital admission for SBO brings the added risk of alcohol withdrawal syndrome (AWS), a condition that can significantly hinder recovery and worsen overall outcomes if not managed effectively. Symptoms of AWS can range from mild anxiety and tremors to severe autonomic dysregulation, seizures, and delirium tremens. These complications can increase the physiological stress on the patient, potentially leading to further liver decompensation and worsening SBO. Implementing the Clinical Institute Withdrawal Assessment for Alcohol (CIWA) protocol allows healthcare providers to closely monitor withdrawal symptoms and provide timely benzodiazepine therapy, such as lorazepam, to mitigate these symptoms safely [10].

In patients with hepatic dysfunction, however, benzodiazepines must be dosed cautiously, as the liver’s reduced capacity to metabolize these drugs increases the risk of sedation and respiratory depression. The combination of withdrawal management, careful dosing, and consistent monitoring through CIWA is essential to balancing effective treatment while avoiding excessive sedation. Furthermore, thiamine and folate supplementations are crucial to prevent Wernicke’s encephalopathy, a neurological disorder often seen in chronic alcohol users with poor nutritional intake, which could further complicate the clinical picture [8].

Nutritional management and risk of malnutrition

The patient’s nutritional status is also a vital consideration in managing SBO, alongside hepatic dysfunction. Chronic alcohol use disorder often leads to malnutrition, as alcohol disrupts normal digestive and metabolic processes, leading to deficiencies in essential nutrients, including proteins and vitamins [8]. In patients with liver disease, malnutrition is associated with worsened prognosis, as it impairs immune function, weakens muscle mass, and diminishes the body’s ability to recover from acute medical issues such as SBO. A comprehensive nutritional assessment and individualized intervention plan, involving a nutritionist or dietitian, are therefore necessary to provide adequate caloric intake, improve immune function, and support hepatic and gastrointestinal healing.

In this case, nutritional support may involve initiating enteral or parenteral nutrition to ensure the patient’s energy needs are met, and the risk of malnutrition is minimized. Vitamins, particularly thiamine, folate, and fat-soluble vitamins (A, D, E, and K), are crucial for patients with ALD, as these patients are often deficient and require supplementation for optimal recovery [20].

Preventing recurrence and long-term management

Long-term management of patients with hepatic dysfunction and SBO must address the root cause: alcohol use disorder. Recurrence of SBO or liver decompensation is likely if alcohol use continues, as alcohol directly contributes to further liver damage and gastrointestinal motility issues. Referral to addiction services, counseling, and rehabilitation support are therefore essential elements of discharge planning. These interventions help prevent recurrence and provide the patient with resources for sustained recovery. Establishing a strong support system and involving family members, when appropriate, can further aid in reducing the likelihood of relapse and improving long-term outcomes.

## Conclusions

Managing SBO in patients with concurrent hepatic dysfunction and alcohol use disorder presents a complex clinical challenge that demands a coordinated, multidisciplinary approach. This case underscores the importance of collaboration among surgeons, gastroenterologists, hepatologists, and nutritionists to effectively manage acute symptoms while addressing the systemic effects of chronic alcohol use.

Hepatic dysfunction alters drug metabolism, immune response, and gastrointestinal motility, necessitating cautious treatment strategies that prioritize infection control, nutritional support, and withdrawal management. Addressing the root cause (alcohol use disorder) through counseling and rehabilitation is essential for preventing recurrence. Ultimately, a holistic, patient-centered care model is vital for improving both immediate outcomes and long-term health in medically complex individuals.
